# Phonological working memory is adversely affected in adults with anorexia nervosa: a systematic literature review

**DOI:** 10.1007/s40519-022-01370-1

**Published:** 2022-02-08

**Authors:** Amelia D. Dahlén, Santino Gaudio, Helgi B. Schiöth, Samantha J. Brooks

**Affiliations:** 1 Section of Functional Pharmacology, Department of Neuroscience, Uppsala University, 75124 Uppsala, Sweden; 2 Department of Biomedicine and Prevention, University of Rome Tor Vergata, 00133 Rome, Italy; 3 Institute of Translational Medicine and Biotechnology, I. M. Sechenov First Moscow State Medical University, Moscow, Russia; 4 School of Psychology, Faculty of Health, Liverpool John Moores University, Liverpool, UK; 5 Neuroscience Research Laboratory (NeuRL), Department of Psychology, School of Human and Community Development, University of the Witwatersrand, Johannesburg, South Africa

**Keywords:** Working memory, Eating disorder, Phonological loop, Visuospatial sketchpad, Anorexic voice

## Abstract

**Purpose:**

Cognitive restraint has potentiating and deleterious effects on working memory (WM) in anorexia nervosa (AN). Conflicting evidence may be due to heterogeneity of tasks examining different WM components (e.g., verbal/auditory versus visuospatial), and differences in adolescent versus adult AN. Additionally, differential cognitive profiles of restricting versus binge/purging subtypes, comorbid psychiatric disorders and psychotropic medication use may confound findings.

**Methods:**

To address these conflicts, 25 studies, published between 2016 and 2021, investigating WM in children, adolescents and adults with AN were systematically reviewed using PRISMA guidelines.

**Results:**

In 71% of WM tasks, no difference in performance between AN patients and age-matched controls was reported, while 29% of WM tasks showed worse performance. Adults with AN displayed deficits in 44% of the verbal/auditory tasks, while performance remained unaffected in 86% of visuospatial tasks.

**Conclusion:**

Examining age groups and WM subsystems separately revealed novel findings of differentially affected WM components in AN. Comorbidities and psychotropic medications were common among AN participants and should be regarded as critical confounding factors for WM measures. Future studies examining different components of WM, acknowledging these confounding factors, may reveal specific deficits in AN to aid treatment improvement strategies.

**Level of evidence:**

I, systematic review.

**Supplementary Information:**

The online version contains supplementary material available at 10.1007/s40519-022-01370-1.

## Introduction

Anorexia nervosa (AN) is an eating disorder (ED) characterised by self-induced emaciation, distorted body image and fear of gaining weight [1]. It also involves strict monitoring of food intake to control body shape, so-called cognitive restraint [2]. The disorder typically emerges in adolescent females and is known for its unremitting treatment resistance throughout the individual’s lifetime [3, 4]. Alongside the medical complications elicited by long-term starvation, AN patients have a high risk of developing affective disorders, personality disorders and substance use disorders [5]. The physical and psychological deterioration in AN tragically causes the highest mortality rate of all psychiatric disorders [6].

Although the aetiology of AN remains largely unknown, a high degree of cognitive restraint coinciding with perfectionism, rigid thinking styles and obsessive–compulsive cognitions generally set AN apart from other EDs, such as Bulimia Nervosa (BN) and Binge Eating Disorder (BED) [7, 8]. However, it is important to note that an individual’s diagnosis may change over the course of illness—often referred to as *trans-diagnostic* [9, 10]. There are several cognitive theories regarding how excessive cognitive control is preserved. One such hypothesis is that variation in working memory (WM) capacity contributes to the obsessive, ruminative and inflexible thought patterns that appear to underlie fluctuating cognitive deficits, appetite restraint and body image distortion in restrictive AN (AN-R) [11–14]. A milder degree of restraint may explain the binge-purging subtype of AN (AN-BP) and the progression into binge-purge and bulimia symptoms, where weight-compensatory behaviours are interleaved with bouts of impulsive and excessive food intake [7].

WM is a crucial executive function responsible for holding and manipulating verbal, auditory and visuospatial information after it is no longer present as a cue [15, 16]. This enables future goals to be achieved despite challenge from distractions [16]. Baddeley and Hitch (1974) postulate that verbal and auditory information are processed by a compartment referred to as the phonological loop (e.g., an *inner voice*) and that visual information is processed by the visuospatial scratchpad (e.g., contributing to body image). These separate subsystems work in concert with the central executive coordinating system [15]. Unlike the single-component visuospatial sketchpad, the phonological loop can be further separated into the phonological store and the articulatory rehearsal process [17]. The former holds speech-based information for approximately 2 s, whereas the latter prevents subsequent decay through subvocal speech rehearsal [17]. Against this background, tasks utilising the phonological loop, e.g., story recall, may give rise to different results than tasks engaging the visuospatial domain, e.g., Rey Complex Figure Task [18].

In individuals with AN, WM is suggested to be disproportionately engaged in negative ruminations about the self, past negative emotional experiences and preoccupations with food and eating [19]. While an excessive activation of WM processes associated with appetite restraint in AN has been suggested to improve overall WM performance in patients [13], a pathological preoccupation with AN-related cues could also have detrimental effects on WM tasks [20]. The potential role of WM processes in the development and maintenance of AN parallels the notion of a controlling internal dialogue first described by Bruch in 1978 [21] and later referred to as the *anorexic voice* [22, 23]. An estimated 96% of individuals with ED reportedly experience this critical inner voice, which is increasingly perceived as controlling throughout the duration of AN [24, 25]. This supports the concept of a verbal cognitive restraint framework for the disorder, where weight is lost through a compulsive adherence to fasting, purging and excessive exercise regimens, which is extremely difficult to alter during clinical intervention [26].

Over the past two decades, there have been several publications exploring altered WM capacity as a cognitive hallmark of AN. Between 2002 and 2015, 45% of the studies reported an enhanced WM performance in AN cohorts, 18% reported deficits while the remaining 37% reported no statistically significant difference from healthy controls [14]. A year later, six reviewed studies examining WM in AN suggested deficits or equal WM performance in AN compared to controls [26]. Given the involvement of WM in cognitive inhibition, goal-orientation and other executive functions associated with core deficits in AN, factors that may confound the findings, such as participant age, disorder subtypes, illness duration, comorbidities and the type of WM tasks used, should be considered as contributing to discrepancies in research findings [28–30].

The purpose of the current review is to systematically examine the literature on WM capacity in AN, published between 2016 and 2021, to provide an updated and more nuanced perspective on the role of WM in AN. Considering possible confounding factors that can affect WM performance, this review aims to provide a more focused account of (a) the specific memory tasks used in line with different subcomponents of WM, i.e. phonological and visuospatial measures, (b) the age group of participants, (c) the AN subtypes included and (d) any psychiatric co-morbidities and medications present in the participant samples.

## Methods

**Fig. 1:**
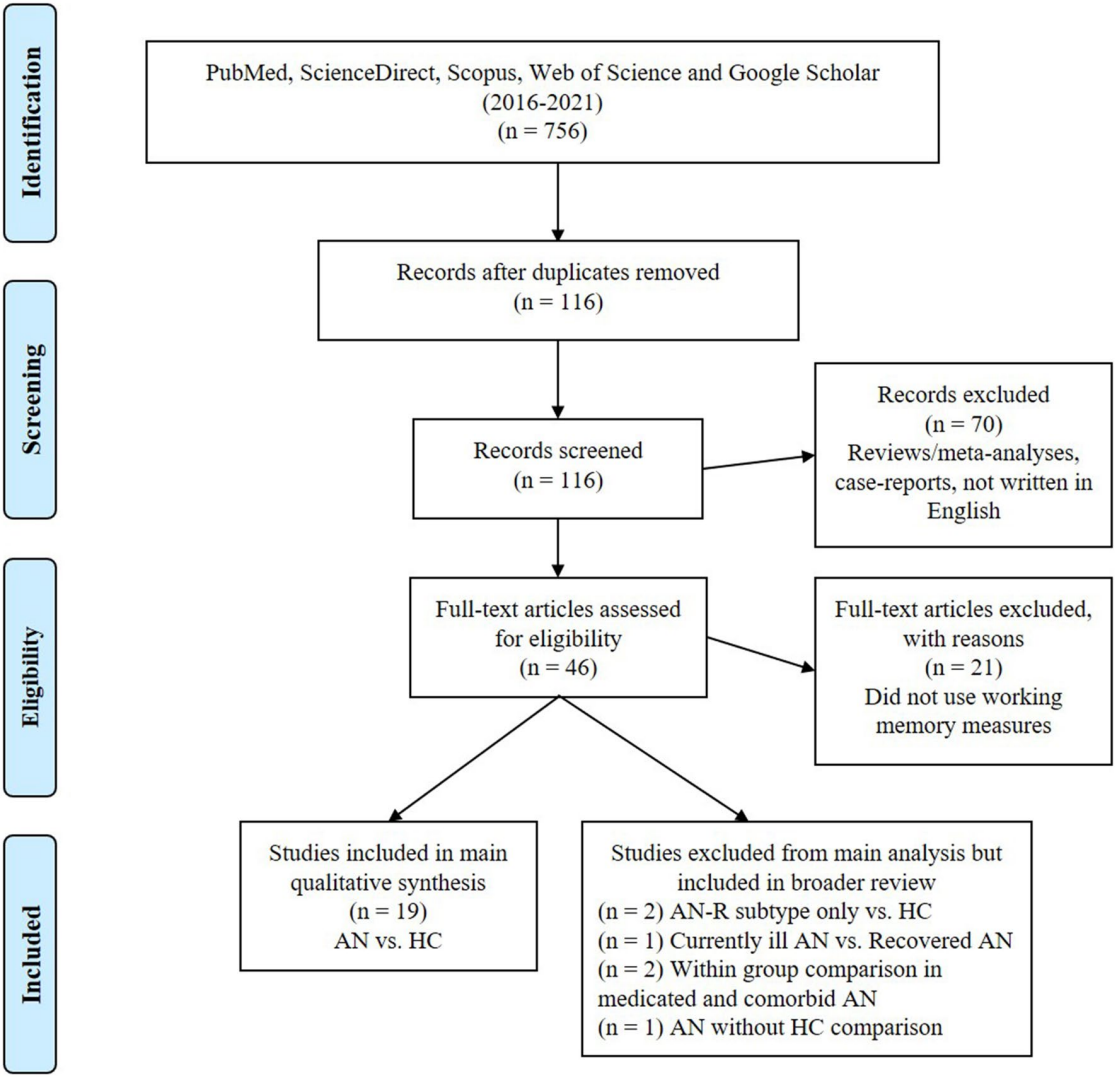
PRISMA Flow Diagram of the relevant steps for the literature search

This review followed the Preferred Reporting Items for Systematic Reviews and Meta-Analysis (PRISMA) guidelines [31] (Fig. 1). The PRISMA checklist of recommended items to be reported can be found in Online Resource Table S1.

### Search strategy and inclusion criteria

For the literature search, PubMed, ScienceDirect, Scopus, Web of Science and Google Scholar were employed. The publication time-frame was limited to January 2016–October 2021 (from the end of the previous review search period, Brooks et al. (2017) [26], to the present) and the following search terms were used in each database search: *anorexia AND working memory; anorexia nervosa AND working memory; anorexia nervosa AND working memory AND cognitive control; anorexia nervosa AND cognitive control; anorexia nervosa AND memory.* The reference lists of the studies were inspected and skilled colleagues within the AN field were consulted for additional relevant papers. The inclusion criteria were peer-reviewed papers that were (1) written in English, (2) included participants with current or recovered diagnosed AN, (3) utilised a minimum of one WM measure, (4) published in peer-reviewed journals. Exclusion criteria were (1) articles not in English, (2) systematic reviews/meta-analysis, book chapters, and perspective articles, (3) research articles not investigating WM, (4) case reports. Authors of eligible primary studies were contacted to obtain additional information if poorly reported.

### Quality assessment and data abstraction

To prevent selection bias, the PRISMA recommendations for systematic literature analysis have been followed and two authors (A.D.D. and S.J.B.) selected studies based on the broad search terms. Risk of bias in individual studies was assessed using the AXIS appraisal tool [32] (Online Resource Table S2). The following characteristics were extracted: type of WM measures used, sample size of ED and healthy control (HC) participants, age, body mass index (BMI), illness duration, subtype of AN, co-occurring psychopathology and medications (Tables 1, 2). The extracted WM tasks were categorised according to the verbal/auditory or visuospatial domain (Online Resource Tables S3, S4). As the aim was to provide a review of variables that may influence WM in AN, factors, such as AN subtypes, psychiatric comorbidities and pharmacological interventions, were not excluded. The heterogeneity of the obtained publications prevented a meta-analysis to be conducted.

**Table 1:** Demographics of participants with anorexia nervosa in the reviewed publications

Study	Nationality	Male/Female M/F	Healthy Controls (HC)			Patients				Eating disorder subtypes (no. of subjects)				Additional details
			Number of HC	Mean age (yrs)	Mean BMI (kg/m^2^)	Mean age (yrs)	Mean BMI (kg/m^2^)	Illness duration (yrs)	In/outpatient status	AN	AN-R	AN-BP	Mixed ED	Comorbidities and medications
Children														
van Noort et al. [39]	German	M/F	30	11.62	18.44	12.17	14.39	0.84	N.R	30	28	2		
Adolescents														
Kjærsdam Telléus et al. [41]	Danish	M/F	60	14.70	N.R	14.70	15.70	1.20	N.R	60	53	7		Anxiety *n* = 13, affective dis., *n* = 14, ASD *n* = 4, psychotic symptoms *n* = 4, meds. *n* = 7
van Noort et al. [39]	German	M/F	30	16.20	21.27	15.93	15.87	0.87	N.R	30	22	8		
Malagoli et al. [37]	Italian	F	124	17.40	N.R	17.90	N.R	N.R	N.R				13	
Vicario and Felmingham [74]	Australian	F	21	15.32	21.11	15.43	16.1	N.R	30 (in)	30				
Zegarra-Valdivia & Chino-Vilca [43]	Peruvian	N.R	15	15.67	N.R	16.27	Not N.R	3.00	N.R	15	4	7	4	
Kucharska et al. [73]	Polish	F	50	16.62	22.55	16.46	14.84	N.R	47 (in)	47	39	8		
Adults														
Terhoeven et al. [61]	German	F	30	24.60	22.83	26.00	14.54	9.30	27 (in)	27	19	8		
Tseng et al. [36]	Taiwanese	M/F	No HC	–	–	27.97	15.5	N.R	N.R	41			227	Depression *n* = 32, bipolar dis. *n* = 9, meds. *n* = 13
Harper et al. [35]	American	F	No HC	–	–	28.20	17.54	N.R	N.R	17				
Cavalera et al. [38]	Italian	F	76	22.46	N.R	24.50	N.R	N.R	37 (in)	37				
Gagnon et al. [57]	French-Canadian	F	23	25.91	21.47	30.80	19.55	N.R	N.R	10	5	5		
Brockmeyer et al. [58]	German	F	40	23.98	21.26	23.73	16.80	N.R	N.R	40	23	17		Depression *n* = 30, anxiety *n* = 2
Adults														
Malagoli et al. [37]	Italian	F	124 17.40 (same HC as adole.)		N.R	28.50	N.R	N.R	N.R				13	Meds. *n* = 14
Rylander et al. [72]	American	F	10	35.00	24.00	33.00	12.70	N.R	N.R	39	20	19		
Konstantakopoulos et al. [59]	Greek	F	42	26.00 (median)	20.70 (median)	26.00 (median)	16.00 (median)	5.00 (median)	46 (out)	46	30	16		
Konstantakopoulos et al. [60]	Greek	F	42	26.00 (median)	21.70 (median)	26.00 (median)	15.50 (median)	5.00 (median)	46 (out)	46	29	17		Meds. *n* = 11
Seidel et al. [42]	Danish	M/F	36	25.42	23.14	28.62	17.31	9.19	26 (in)	26				Mood dis. *n* = 9, stress-related dis. *n* = 12, personality dis. *n* = 4, Meds. *n* = 16
Ogata et al. [34]	Japanese	F	10	32.40	18.82	32.17	12.65	N.R	N.R	12	12			
Terhoeven et al. [58]	Greek	F	30	23.70	21.20	23.20	15.10	6.10	28 (in) 1 (out)	29	21	8		
Adults and adolescents grouped														
Øverås et al. [75]	Norwegian	F	34	18.62	N.R	18.75	N.R	N.R	N.R	36				
Cipolletta et al. [71]	Italian	F	12	23.04	19.88	23.04	16.86	N.R	N.R	12				
Foerde and Steinglass [40]	American	M/F	26	22.90	21.60	24.70	16.80	N.R	N.R	36	19	17		Depression *n* = 3, anxiety *n* = 7, meds. *n* = 5
Natalia et al. [56]	Swiss	F	20	20.10 (median)	21.30 (median)	20 (median)	16 (median)	4.00	14 (in) 15 (out)	29	23	6		
Tamiya et al. [62]	Japanese	F	69	34.36	N.R	29.10	15.53	N.R	16 (in) 24 (out)	40	22	18		Meds. *n* = 12
Adults and adolescents grouped														
Cholet et al. [33]	French	F	59	21.30	21.10	20.70	15.20	3.50	N.R	59	59			Anxiety *n* = 34, affective dis. *n* = 21, ADHD *n* = 4, addictive behaviours *n* = 6, suicide risk *n* = 26, meds. *n* = 40
Tenconi et al. [76]	Italian	F	58	19.50	21.30	19.60	15.60	1.99	N.R	56				
Overall means and sums			947	22.20	21.40	22.97	15.71	4.00	225 (in) 188 (out)	850	428	163	257	

Attention deficit hyperactivity disorder (ADHD), autism spectrum disorder (ASD), binge/purging subtype of anorexia nervosa (AN-BP), body mass index (BMI), disorder (dis.), restricting subtype of anorexia nervosa (AN-R), medications (meds.), mixed eating disorders (ED): anorexia nervosa (AN), binge-eating disorder (BED), bulimia nervosa (BN), Eating Disorder Not Otherwise Specified (EDNOS), not reported (N.R.), Other Specified Feeding or Eating Disorder (OSFED)

**Table 2:** Descriptions of working memory tasks utilised by the studies in this review

Battery/Author	Test	Assesses	Description
Phonological loop—verbal and auditory WM			
WAIS-R, III, IV Wechsler [44–46]	Arithmetic	Auditory WM, active mental manipulation, quantitative reasoning, arithmetic abilities	Verbal presentation of increasingly difficult arithmetic word problems. Elements need to be stored until all information is given, before a response is formulated
WAIS-R, III, IV WMS-III Wechsler [44–47]	Digit span	Uses clustering **Forward:** maintenance, short-term memory **Backward:** auditory WM, mental manipulation **Sequencing:** auditory WM, more active form of mental manipulation	Experimenter verbalises a number sequence to be repeated by the participant: -**Forward:** in the same order -**Backward:** in reverse order. Verbal responses are initiated and rehearsed as soon as the first digit is presented **-Sequencing:** ascending order. Verbal response cannot be rehearsed until the last digit is presented
WAIS-III, IV Wechsler [45, 46]	Letter Number Sequencing (LNS)	Complex auditory WM, requires reordering, storage and retrieval	Verbal presentation of a letter-number sequence to be reported back in alphabetical and ascending numerical order
CogTrack Wesnes et al. [53]	Immediate Word Recall	Verbal WM	15 words presented on a screen, 1 word every 2 s. The subject is given one minute to type all words that can be recalled
CogTrack Wesnes et al. [53]	Numeric working memory	Active mental manipulation	A combination of 5 digits are presented on the screen, 1 every 1.2 s. This is followed by a 30 probe digits. The subject has to indicate whether or not the probe digit was in the original series, using keyboard arrows
Rey [48]	Rey Auditory Verbal Learning Test (RAVLT)	Short-term auditory-verbal memory	Free-recall of a 15-item list tested over five trials, then an interference 15-item list is presented. Participants need to repeat the original list after interference and again after 30 min, followed by a yes–no word recognition trial
Daneman & Carpenter [51]	Reading Span task (RSPAN)	Verbal WM	Recall of a letter sequence that appears on a screen together with a phrase. Participants read the sentence aloud and judge if it makes logical sense. When presented with a recall cue, the letters from the previous sets must be recalled in the correct order
Wechsler Memory Scale (WMS-R) Wechsler [52]	Story recall	Auditory-verbal WM, logical memory	Two neutral stories are read aloud to participants, which have to be recalled shortly after hearing the story (immediate recall) and after 30 min (delayed recall)
Kirchner [64]	N-back	Encoding and a temporary storage in WM, continuous updating, and inhibition of irrelevant items from WM	Participants are presented a series of visual stimuli. For each trial, they are asked if the stimulus matches the stimulus n trials prior. E.g. 2-back, 3-back tasks
Visuospatial Sketchpad			
Corsi [63]	The Corsi Block Tapping Test (Corsi Blocks)	Visuo-spatial WM, spatial attention	Participants reproduce a wooden block tapping sequence, with increasing length (9-cubes), presented by the experimenter. The test ends when the incorrect reproductions exceed the proportion of acceptable errors per sequence length
Osterrieth [66] and Rey [65]	Rey Complex Figure Test (RCFT)	Visual-spatial processing and visual WM	Participants are required to copy a figure containing 18 global and local elements **Immediate recall:** participants reproduce the figure from memory **Delayed recall:** participants reproduce the figure after 20–30 min
WMS-R, III Wechsler [47]	Spatial span Modification of the Corsi Blocks test	Visuo-spatial WM, attention, holding of memory and response formulation	A visual analogue to the Digit Span task, with 10 cubes, instead of 9 in Corsi’s original version. Forward and backward sequences are tested
WAIS Smith [67]	Symbol-Digit Modalities Test (SDMT)	Visuo-spatial WM processing, processing speed and attention Can be adapted to verbal WM	Participants use a symbol-number key to code as many series of symbols with numbers as possible in 90 s
Kane et al. [68]	Symmetry span task (SymmSpan)	Complex WM capacity	Two different tasks performed simultaneously. The first requires the recall of a square sequence presented on a screen. The second involves judging whether figures are symmetrical or not. Thereafter participants are to recall the whole sequence of squares in the correct order
Armitage [69] and Reitan [70]	Trail Making Test (TMT)- part A and B	Visual perception, visual-motor coordination, and visual-spatial operational memory	Test consisting of matching circles with numbers and letters **Part A:** measures psychomotor speed and visual-motor coordination **Part B:** measures the skills in part A and evaluates visual-spatial operated memory and executive functions **Condition 4:** corresponds to Part B
CogTrack Wesnes et al. [53]	Spatial working memory	Visuo-spatial WM, attention, holding of memory and response formulation	3 by 3 array of light bulbs is presented on the screen for 10 s. The subject must memorise the position of four lit bulbs. This is followed by 36 presentations of the 3 by 3 array, with a single bulb lit. The subject is instructed to decide whether or not the lit bulb was lit in the original presentation

## Results

The literature search yielded 25 studies examining WM capacity in AN across 14 countries, 19 of which were case versus control studies and formed the main analysis. The remaining six studies examined WM within AN-R [33, 34], pre-and post-recovered AN [35], comorbid and medicated ED [36, 37] or did not perform statistical comparisons between the clinical ED and non-clinical HC groups [38] (Table 3). The combined sample size included 850 AN participants with a mean age of 23 years and a mean BMI of 15.7. The patient sample sizes of individual studies ranged from *n* = 10 to *n* = 60. The combined HC sample size was 947 with a mean age of 22.2 years. BMI values for the HC were not consistently reported but for the 18 studies that did, the mean BMI was 21.4 (Table 1). 19 of the studies exclusively included female participants, with the exception of the mixed gender groups in five studies [36, 39–42]. Zegarra-Valdivia and Chino-Vilca [43] did not specify the genders of their sample. Of the ten publications which reported patient status, 225 were inpatients while 188 were outpatients. The subsequent sections will elaborate on 1) memory performance across tasks, 2) differential WM performance according to age groups, 3) WM in the subtypes of AN, 4) psychiatric comorbidities and 5) psychotropic medications among the AN participants.

**Table 3:** Overview of the working memory modalities and sample sizes of the reviewed studies

Both verbal/auditory and visuospatial (Total *n* = 431, AN, *n* = 197, HC, *n* = 234)	Verbal/auditory (Total *n* = 286, AN, *n* = 147, HC, *n* = 139)	Visuospatial (Total *n* = 585, AN, *n* = 275, HC, *n* = 310)
Main analysis of case versus control studies		
Terhoeven et al. [61]	Zegarra-Valdivia & Chino-Vilca [43]	van Noort et al. [39]
Foerde and Steinglass [40]	Brockmeyer et al. [58]	Kjærsdam Telléus et al. [41]
Natalia et al. [56]	Konstantakopoulos et al. [59]	Cipolletta et al. [71]
Tamiya et al. [62]	Konstantakopoulos et al. [60]	Øverås et al. [75]
Gagnon et al. [57]		Vicario and Felmingham [74]
Seidel et al. [42]		Kucharska et al. [73]
Terhoeven et al. [58]		Rylander et al. [72]
		Tenconi et al. [76]

Both verbal/auditory and visuospatial (Total *n* = 255, ED, *n* = 72, HC, *n* = 183)	Verbal/auditory (Total *n* = 176, ED, *n* = 90, HC, *n* = 86)	Visuospatial (Total *n* = 17, ED, *n* = 17)
Studies included in the narrative review		
Malagoli et al. [37] (medicated ED group)	Tseng et al. [36] (ED group with comorbid psychiatric disorders)	Harper et al. [35] (pre-and post-recovered AN)
Cholet et al. [33] (AN-R only)	Cavalera et al. [38] (ED group not compared to HC)	
	Ogata et al. [34] (AN-R only)	

Anorexia nervosa (AN), eating disorders (ED), healthy controls (HC), restricting subtype of anorexia nervosa (AN-R)

### General working memory performance across all tasks

**Fig. 2:**
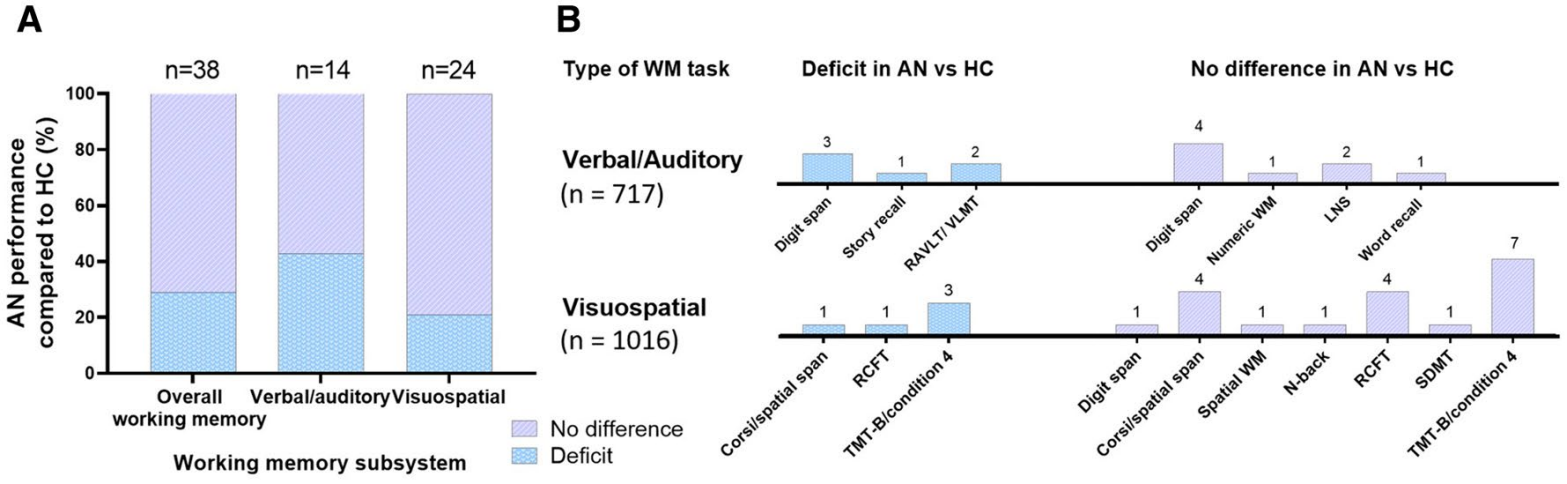
**A** Working memory (WM) performance in the anorexia nervosa (AN) group compared to the healthy control (HC) group in all WM tasks, verbal/auditory and visuospatial WM tasks, respectively. **B** AN performance in specific WM tasks, compared to HC. The total sample size in the verbal/auditory domain was 717 participants, and for the visuospatial domain the total sample size was 1016 participants. The annotated *n* indicates the number of WM tasks used in the reviewed studies. Letter Number Sequencing (LNS), Rey Auditory Verbal Learning Test (RAVLT), Verbal Learning and Memory Test (VLMT), Rey Complex Figure Test (RCFT), Symbol-Digit Modalities Test (SDMT), Trail Making Test (TMT-B)-part B

Sixteen commonly used WM measures were identified (Table 2). Among the 19 publications performing statistical comparisons between AN and HC groups, a total of *n* = 38 independent WM tasks were conducted across all studies. AN participants exhibited impairments in 28.9% of the WM tasks while their performance was unaffected in 71.1% (Fig. 2A).

#### Verbal and auditory domains

These included Arithmetic, verbal Digit span, Letter Number Sequencing (LNS) [44–47], Rey Auditory Verbal Learning Test (RAVLT) [48] (and its German equivalent the Verbal Learning and Memory Test (VLMT) [49, 50], Reading Span task (RSPAN) [51], Story recall [52], Numeric WM and Immediate Word Recall [53] (Online Resource Table S3). AN and HC groups were compared in *n* = 14 verbal/auditory tasks, with a total sample size of *n* = 717 (Fig. 2). The scores from Arithmetic and Digit span subsets of the WAIS-IV battery were summed to create a Working Memory Index (WMI) [54]. The backwards verbal digit span was the most recurring measure utilising the phonological loop. Of the studies directly comparing AN and HC cohorts in the verbal digit span, 57% of the studies did not detect any significant difference between the groups [55–58], while 43% found impairments in the AN participants [43, 59, 60] (Fig. 2B). Zegarra-Valdivia and Chino-Vilca [43] also highlighted a significant correlation between illness duration and poorer performance in the verbal/auditory digit span. Cavalera et al. [38] did not perform direct statistical comparisons between AN and HC groups, but put forward the notion that the feeling of shame, elicited by writing about an unpleasant experience in between digit span tests, has a stronger negative effect on performance in AN, BN, and Other Specified Feeding or Eating Disorder (OSFED) populations than in HC. Moreover, Terhoeven et al. [56] and Natalia et al. [61] both presented immediate recall deficits in RAVLT/VLMT for the AN group. Furthermore, Terhoeven et al. [61] measured significantly worse performance among AN participants in the immediate story recall. For the LNS, numeric WM and immediate word recall no significant differences between the AN and HC samples were detected [40, 42, 62]. Overall, the AN population had worse WM performance in 42.9% of the verbal/auditory tasks, when compared to HC, and performed at comparable levels to HC in the remaining 57.1% (Fig. 2A).

#### Visuospatial domains

These included, the Corsi Block Tapping Test [63] (and its modification the Spatial span [47]), visuospatial Digit span [44–47], N-back [64], Rey Complex Figure Test (RCFT) [65, 66], Symbol-Digit Modalities Test (SDMT) [67], Symmetry span task (SymmSpan) [68], Trail Making Test (TMT) [69, 70], and Spatial WM [53] (Online Resource Table S4). AN and HC groups were compared in *n* = 24 visuospatial tasks, with a total sample size of *n* = 1016 (Fig. 2). When measuring visuospatial WM, the TMT was the most common task, followed by the Corsi Blocks/Spatial span and the RCFT (Online Resource Table S4). AN and HC groups tended to perform at similar levels across the three measures with no significant difference found in 70% of the studies using the TMT, 80% of the Corsi blocks/Spatial span studies and in 80% of the RCFT studies [39–41, 56, 57, 61, 62, 71, 72, 74–76] (Fig. 2B). Natalia et al. [56] reported worse performance for individuals with AN in the backwards condition of the Corsi Blocks/Spatial span and in the TMT. Similarly, Kucharska et al. [73] and Terhoeven et al. [58] found TMT impairments in their AN sample compared to the HC. In contrast, no significant difference emerged in the visuospatial digit span [74], spatial WM task [42] n-back or SDMT [56]. The currently ill AN (AN-CC) group of Harper et al. [35] demonstrated an impaired TMT-B performance in comparison to recovered AN (AN-CR). However, the groups did not differ in the RCFT, and were not compared to a HC group. Across the reviewed studies directly comparing AN and HC samples, AN participants were impaired in 20.8% of the visuospatial WM tasks but showed normal WM capacity in 79.2% (Fig. 2A).

### Differential WM performance according to age groups

Out of the reviewed studies, one investigated early-onset AN (EO-AN) in children (9–14 years), six tested adolescents (11.5–19 years), thirteen tested adults (> 18 years) and seven studies used a mixed participant group with both adolescents and adults (Table 1).

#### Children with EO-AN

EO-AN childern did not differ from age-matched HC in visuospatial WM abilities measured in the RCFT and TMT [39]. Nor did the children with EO-AN and adolescents with AN differ in the TMT, while healthy adolescents were significantly better than healthy children in the sample [39].

#### Adolescents with AN

**Fig. 3:**
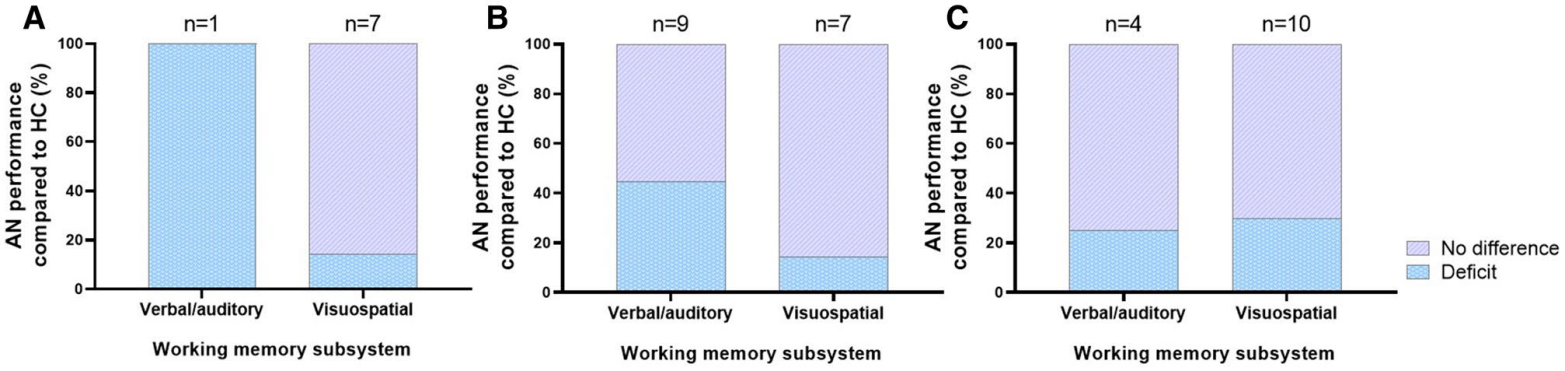
Working memory (WM) performance in adolescent (**A**), adult (**B**) and mixed aged (**C**) anorexia nervosa (AN) groups compared to age-matched healthy control (HC) groups in verbal/auditory and visuospatial WM tasks. The annotated *n* indicates the number of WM tasks used in the reviewed studies

Were also found to have deficits in the visuospatial TMT [73]. The remaining 86% of the studies using visuospatial digit span, RCFT and TMT in adolescent AN did not reveal statistically significant differences, as illustrated in Fig. 3A [39, 41, 74]. Zegarra-Valdivia and Chino-Vilca (2018) were the only research group to report an inferior verbal/auditory WM performance in adolescent AN participants completing the verbal digit span task (Online Resource Table S3) [43].

#### Adult individuals with AN

Performed worse than HC in 44.4% of the verbal/auditory tasks [59–61], while no difference was found in 55.6% [42, 55, 57, 58] (Fig. 3B). The findings in the visuospatial WM domain showed no prominent differences between the AN and HC cohorts in 85.7% of tasks [57, 61, 72, 75], while the AN group was worse than the HC in the remaining 14.3% [58].

#### Studies with a combination of adolescents and adults

No WM component differences were found at the group level when age groups of AN cohorts were mixed in studies. The reviewed studies presented WM impairments in approximately 25% of the tasks relying on the phonological loop and in 30% using the visuospatial sketchpad [56, 76] (Fig. 3C). No statistically significant differences emerged between mixed-age AN and HC in 75% of the verbal/auditory WM tasks [40, 56, 62] or in 70% of the visuospatial WM tests [40, 56, 62, 71] (Fig. 3C).

### WM in the subtypes of anorexia

Patient subtype specifications were collected from sixteen of the reviewed publications, giving a total *n* = 428 for AN-R and *n* = 163 for AN-BP. The reviewed studies gave mixed accounts regarding potential subtype-specific changes in WM capacity, presented in more detail below.

#### Restricting AN patients

**Fig. 4:**
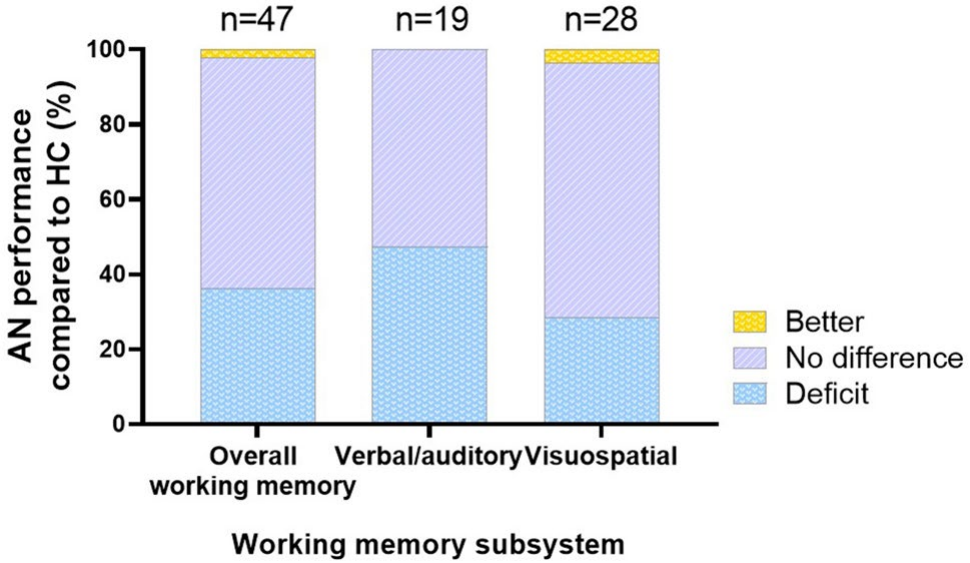
Working memory (WM) performance in the full anorexia nervosa (AN) group, with and without reported AN subtypes, psychiatric comorbidities and medications, compared to the healthy control (HC) group in all WM tasks, verbal/auditory and visuospatial WM tasks, respectively. The annotated *n* indicates the number of WM tasks used in the reviewed studies

in the mixed adolescent and adult group of Natalia et al. [56] presented with significantly impaired performance in the verbal/auditory VLMT, and the visuospatial backward Corsi Blocks, SDMT and in the reaction time of the 2-back test [56]. Interestingly, AN-R did not deviate from the HC in forward Corsi Blocks, digit span, 3-back or the RCFT. Nor did the adult and mixed adolescent and adult groups of AN-R patients show altered WM functioning in the verbal/auditory digit span and LNS in comparison to HC [33, 34, 55, 62] or in the visuospatial spatial span [62]. However, adolescents and adults with AN-R combined had a significantly faster recall time than the control group for the visuospatial RCFT [33]. This was an outlier study responsible for the superior AN-related performance in 4% of the visuospatial WM tasks (Fig. 4, Online Resource Table S4). The recent publication by Rylander et al. (2020) investigated cognitive functioning in patients with severe AN pre- and post-medical stabilisation [72]. Severe AN was defined as weighing less than 70% of the ideal body weight (IBW), calculated using patient height and a goal BMI (IBW (kg) = BMI × height^2^) [77]. Neither AN-R nor AN-BP patients differed from the non-ED control group in baseline visuospatial TMT results. After three weeks of refeeding and improved organ and oedema status, AN-R displayed significant improvements in TMT scores while the AN-BP patients did not.

#### Binge/purging AN patients

were argued by Tamiya et al. [62] to have more widespread cognitive dysfunctions, but this was not evident in the WM measures [62]. Individuals with AN-BP performed at the same level as HC in the verbal/auditory digit span and LNS, as well as the visuospatial spatial span [55, 62]. The small AN-BP sample sizes were insufficient to perform additional statistical analysis in Natalia et al. [56] and Terhoeven et al. [58].

### Psychiatric comorbidities among the AN participants

In order of prevalence, the following comorbidities were reported: depression [36, 40, 55], anxiety [33, 40, 41, 55], affective disorder [18, 33], suicide risk [33], stress-related disorders, mood disorder, personality disorder [42], bipolar disorder [36], addictive behaviours, attention deficit hyperactivity disorder [33], autism spectrum disorder and psychotic symptoms [18] (Table 1). Obsessive compulsive disorder (OCD) was also present in the patient cohorts of van Noort et al. [39] and Malagoli et al. [37], but no exact demographic values were given and no links between WM performance and OCD were made despite the high comorbidity risk in AN, and in particular AN-BP [78, 79]. Twelve of the studies included in this review outlined certain psychiatric co-morbidities and psychotropic medications as exclusion factors in AN patient recruitment [34, 55, 57–62, 72–74, 76].

In Tseng et al. [36] mixed ED group (AN, BN, BED, and Eating Disorder Not Otherwise Specified (EDNOS)), the participants with comorbid major depressive disorder (MDD) and bipolar I or II disorder were significantly worse in verbal/auditory arithmetic and digit span tasks, than ED patients with just MDD. Moreover, co-occurring bipolar I disorder was associated with overall impairments in WM and intelligence measures. Although the patients with AN in Konstantakopoulos et al. [60] lacked a comorbid psychiatric diagnosis, they found that those suffering from intense body image delusions performed significantly worse than the non-delusional patients in the verbal/auditory digit span task.

### Psychotropic medications used in the AN sample

Eight of the reviewed publications included information on psychotropic medication use in AN participants of both subtypes [33, 36, 37, 41, 60–62, 73] (Table 1). Medications included antidepressants (e.g., selective serotonin reuptake inhibitors), anxiolytics, antipsychotics/neuroleptics and benzodiazepines. Medicated and unmedicated participants in Terhoeven et al. [61] did not differ regarding WM capacity. Conversely, Malagoli et al. [37] showed that a combined use of antidepressants and antipsychotics was related to significantly lower scores in the verbal/auditory RSPAN and visuospatial SymmSpan. When psychiatric comorbidities, medications and AN-R-specific data were visualised together with above AN versus HC results, AN-related deficits in occurred in 47.4% of the phonological tasks (Fig. 4). Taking the comorbidities, psychotropics and AN-R results into account also diminished the percentage of unaffected performance in visuospatial WM tasks from 79.2 to 67.9%, while AN-related visuospatial deficits increased from 20.8 to 28.6% (Fig. 4).

## Discussion

Earlier reviews on WM functioning in AN have provided various perspectives, ranging from impaired [80], enhanced [14], unaffected [81] to overall inconsistencies [26]. Progressing previous work solely focusing on WM in general, this current review presents novel findings that WM deficits associated with the phonological loop occur in 44% of tasks examining adults with AN versus healthy controls (HC), as opposed to only 14% of tasks relying on the visuospatial sketchpad. This phonological loop deficit was also observed in 47% of studies of AN adults when considering the restricting subtype, psychotropic medication and comorbidities combined. However, when examining mixed adolescent and adult groups, or considering both phonological and visuospatial WM tasks together, no deficits emerge in the AN literature published between 2016 and 2021. Against these new observations, the following sections will discuss in more detail how WM performance may contribute to core symptoms in AN, and the potential implications phonological deficits may have for therapeutic treatment strategies.

### The phonological loop and visuospatial sketchpad are differentially affected in AN

The well-established WM model postulates a division between phonological and visuospatial processing [15], highlighting a need to consider these specific domains separately in AN which has not been met in previous reviews [14, 26, 80–82]. Of the seven studies which included both verbal/auditory and visuospatial tasks, four of them did not detect a significant difference between AN and HC groups in either domain (a combined sample size of 266) (Table 3) [40, 42, 57, 62]. In the other three studies (a combined sample size of 165), there were mixed reports of verbal/auditory WM deficits [61], visuospatial WM deficits [58], and deficits in both WM domains for AN participants compared to HC [56]. By dividing the extracted WM tasks into those that measured verbal/auditory versus visuospatial domains, from all case versus control studies with a total sample size of 1302 participants, it was revealed that the AN group may experience greater difficulties in the verbal/auditory rather than the visuospatial aspects of WM where performance appears largely intact (Fig. 2A).

Similar findings have been reported by Biezonski et al. [83], where patients with AN were disadvantaged in the verbal/auditory LNS task but not the visuospatial TMT, in comparison to HC. Conversely, Lao-Kaim et al. [84] argue that patients with AN use the same cognitive strategies as HC to solve verbal WM tasks, but that visuospatial WM is dysfunctional [80]. A compromised visuospatial sketchpad may be due to intense weight fixation and other body dysmorphic obsessions [20]. Nevertheless, the 24 experiments across seven different visuospatial WM tasks examined in this review do not support inefficient visuospatial WM processing as a cognitive hallmark of AN, as the patient groups did not significantly deviate from the HC even in the review’s more recurring tasks, such as the Corsi Blocks and the TMT (Fig. 2, Online Resource Table S4). In comparison to the visuospatial domain, the verbal/auditory component of WM was understudied in the recent publications, with only 14 experiments across six different verbal/auditory WM tasks comparing a total of 717 AN and HC participants. Despite the overall smaller sample size, in comparison to the 1016 participants tested in visuospatial WM tasks, an intriguing trend of phonological deficits emerged for adult AN patients versus HC in 44% of tasks (Fig. 3B).

As previously mentioned, the phonological loop is reliant on internal speech-mediated rehearsal [17]. Inhibiting rehearsal, e.g., through articulatory suppression by vocalising irrelevant words, may negatively impact WM performance [85]. Buchsbaum [86] stresses the importance of subvocal speech in a sense of self. The ability to hear and listen to this inner voice perceived to be one’s own, assists in short-term recall but also with keeping internal mental representations active in WM. In line with this, AN has been described as a disorder of the self, where the anorexic inner voice is a key psychological component [24, 87, 88]. Indeed, the intrusive internal commentary on body shape, weight and eating has been associated with more harmful eating attitudes and restrictive or compensatory behaviours [24]. Thus, an inner voice preoccupied with AN-salient ruminations may impinge on non-AN-related rehearsal processes, perhaps comparable to articulatory suppression. In turn, the efficiency of verbal/auditory WM may be reduced, as indicated in the reviewed studies (Online Resource Table S3). Inefficient WM phonological rehearsal and updating may also be implicated in the cognitive and behavioural rigidities in AN and may contribute to its self-perpetuating nature [88].

### Testing adolescents and adults with AN separately reveals WM deficits

The neural circuitry in fronto-striatal and parietal regions associated with WM undergoes a substantial maturation throughout adolescence and well into adulthood [89–91]. As AN typically has an adolescent onset, it is of interest to examine how neural development of cognitive control is affected in different age groups of AN patients. The Children with EO-AN did not differ from their healthy counterparts in visuospatial WM, but nor was an expected age-related improvement detected between EO-AN and adolescent AN groups [39]. Children tend to make more errors in WM maintenance and manipulation than adolescents and adults [92]. This has been attributed to a failure to recruit the dorsolateral prefrontal cortex (dlPFC) and superior parietal cortex, which are increasingly engaged with age, as WM processes become more refined [91]. The lack of age-related improvement in van Noort et al. (2016) may be due to the cross-sectional design of the study rather than a longitudinal, but it could also indicate a cognitive disruption during a critical developmental period.

Separate adolescent and adult AN age groups showed similar trends of a largely preserved visuospatial WM. Adolescents with AN were described to have an inferior verbal digit span performance [43], which is in accordance with earlier reports of limited short‐term verbal memory in adolescent AN [18]. However, additional reports of verbal/auditory WM in adolescents were lacking, which prevents further theories regarding differentially affected WM subsystems in adolescent AN. Curiously, the adults with AN were more likely to underperform compared to HC in tasks examining the phonological loop, e.g. RAVLT/VLMT and story recall, than in visuospatial measures [61]. This presents the possibility of a subtype-specific WM deficit in adult AN (Fig. 3B). Although an age-related maturation of fronto-parietal structures is not the sole driver of WM development, adult WM deficits are a cause for concern [90]. With age comes the ability to integrate additional executive networks, e.g., inhibitory control, and deactivate non-executive regions, e.g., the default network, which are critical during high WM loads [93, 94]. With a longer illness duration and accompanying malnutrition, more severe long-term effects on the brain and cognition are anticipated [82, 95, 96]. Regardless, there is a lack of consensus of the impact of illness duration on WM function in the reviewed literature. Zegarra-Valdivia and Chino-Vilca [43] highlighted a significant correlation between illness duration and poorer WM performance, while Tamiya et al. [62] and Malagoli et al. [37] did not find evidence for this. Alternatively, the plasticity of the adolescent brain could be concealing AN-induced difficulties with WM, while the less plastic adult brain may fail to compensate for these alterations and gives rise to more prominent deficits [89, 91].

Patient cohorts with both adolescents and adults demonstrated intact WM functioning in 71% and impaired performance in 29% of all tasks, consistent with Reville et al. [81] review and the claims of a preserved general WM capacity even in the chronically starved state of AN [84, 97]. Unlike what became evident in the separate adolescent and adult groups, distinguishing between the verbal/auditory and visuospatial tasks did not unveil AN-related deficits in either domain (Fig. 3C). What becomes clear from examining the distinct age groups in the reviewed literature, is that separating adolescents and adults could be a prerequisite for disentangling AN-related alterations in WM. Thus, future studies could examine age ranges that reflect different stages of development to determine the influence of neuronal development differences and duration of illness on WM performance.

### How does WM differ in subtypes of AN?

According to the impulse control spectrum model of EDs, individuals with AN-R have a higher degree of cognitive restriction compared to those with AN-BP who engage in binge-eating and weight-compensatory behaviours [7, 26]. On the other hand, patients with AN-BP have been shown to be more perfectionistic than those diagnosed with AN-R [98]. Against this background, the reviewed publications were examined for subtype-specific alterations in WM. The restricting subtype was found to be more commonly studied than binge-purge AN in the combined sample. Cholet et al. [33] proposed that the AN-R classification is further divided into two profiles, (1) more extensive cognitive impairment and milder psychopathological symptoms and (2) symptoms of depression and anxiety and less affected in the cognitive domain. Surprisingly, it was the first subgroup that outperformed HC in visuospatial RCFT recall time which was the only account of an enhanced WM capacity in the reviewed publications [33]. Advantages in RCFT and higher accuracy in the n-back task have been previously reported for AN-R cohorts [13, 18]. However, an elevated performance did not apply to the visuospatial TMT or spatial span WM measures [18]. Thus, deviating WM performances in AN-R may be highly task-dependent.

As previously mentioned in the introduction, an individual’s subtype is not static and may change over the course of illness [9, 10]. Often those diagnosed with AN-R move towards the more impulsive pole of the spectrum model, and into the AN-BP category following treatment [7, 9, 99]. While Tamiya et al. [62] reasoned that the AN-BP subtype had more extensive cognitive dysfunctions in processing speed, attention and problem-solving, Rylander et al. [72] discussed that AN-BP patients might be spared from the chronic nutritional deprivation in AN-R. Still, the relationship between malnutrition, BMI and cognition is complex and requires further investigation [62, 72]. The high degree of cross-over between subtypes also poses the question if they should be considered phases rather than separate categories and should be evaluated in future research [9, 100, 101].

### Comorbidities and medications are confounding factors in the reviewed studies

Psychiatric comorbidities are common in EDs [5] and WM deficits are considered general features of psychotic and affective disorders [102–104]. Comorbid participant samples are indisputably more representative of ED patients, but the intertwined psychopathology patterns make it difficult to unveil disorder-specific WM changes. Therefore, this review examined the occurrence of comorbidity in the tested AN groups. The most prevalent comorbidities were depression and anxiety. This is consistent with the risk loci for major depressive disorder (MDD) and anxiety identified in an extensive genome-wide association study (GWAS) based on 16,992 AN cases [78]. While both psychiatric disorders have been described as disruptive for WM, anxiety may specifically exacerbate EF deficits in EDs [102, 103, 105].

None of reviewed studies propose anxiety as an aggravating factor in AN-related WM impairments. Instead, Tseng et al. [36] presented manic-depressive bipolar I disorder as having adverse effects on WM when co-occurring with EDs and MDD. Although clear associations between bipolar disorder and BN have been made, the relationship with AN is more obscure [106, 107]. Nevertheless, comorbid bipolar and eating disorders may result in an earlier onset and more rapid changes between manic/hypomanic and depressive states [108]. The increased propensity of WM deficits in the more severe subtype bipolar I disorder noted by Tseng et al. [36], may be prompted by white matter abnormalities affecting the dorsal cognitive control system, while white matter abnormalities in hypomanic-depressive bipolar II disorder are more prominent in areas relevant to ventral emotion processing [109, 110]. Likewise, different EDs may have varying psychiatric risks which could be problematic for interpreting results from mixed ED cohorts, such as Tseng et al. [36], Cavalera et al. [38], and Malagoli et al. [37]. Like the aforementioned Harper et al. [35] study, Tseng et al. [36] did not incorporate HC into their methodology which limits the conclusions that can be drawn from these reviewed publications.

In the same manner that psychiatric comorbidities may influence WM performance, the medications taken to treat these may also cause aberrant WM processing, as demonstrated by the ED group receiving antidepressants and antipsychotics in Malagoli et al. [37]. Antidepressants, such as serotonergic reuptake inhibitors, improve WM in MDD patients while potentially having adverse WM effects in healthy subjects [111, 112]. Furthermore, the antipsychotic risperidone has been found to cause long lasting WM impairments in schizophrenia patients [113]. Risperidone antagonises dopaminergic D_2_ and serotonergic 5-HT_2A_ receptors, triggering a downregulation of D_1_ receptors and a reduction of 5-HT_2A_-mediated dopamine release in the dlPFC [114, 115]. While this mechanism of action is beneficial for reducing symptoms of schizophrenia, it may also disrupt dopaminergic signalling involved in WM, whereby both too high and too low levels in the PFC negatively affects performance [116]. Despite the well-established insights on how psychotropic medications alter cognitive processes, only a minority of the reviewed studies perform separate statistical analyses to address this issue. Together with the studies with mixed ED groups, comorbidities and without HC, these limitations further impede conclusions on how AN specifically affects WM.

### Neural perspectives of the WM components in AN

WM is broadly considered a top–down cognitive control process that acts in opposition to bottom–up appetite and salience networks [117, 118]. However, neuroanatomical research is divided in attributing WM processing changes in AN to either excessive dorsal control or alterations in ventral dopaminergic signalling [19, 119–122]. Kaye et al. [26] argue that individuals with AN have a hyperactive top-down modulation which drives the mindset of forgo food now, become thinner later. Alternatively, excessive top–down control could be a reaction to a highjacked bottom–up reward circuitry where anorexigenic behaviours have gained reward salience in a manner resembling addiction [123–125]. As the insula acts as a pivoting point between these two neural processing paths (PFC in top–down and striatum in bottom–up), Nunn et al. [126] offer an additional hypothesis of a rate-limiting dysfunction creating an imbalance between cognition and interoception in the anorectic brain.

In light of the current review’s findings, it is of particular interest to disentangle how the neural pathways of the phonological loop and visuospatial sketchpad are influenced in AN. Verbal/auditory tasks recruit language production and comprehension regions, such as Broca’s area in the frontal lobe, Wernike’s area in the temporal lobe and the supra-marginal gyrus [127]. Visuospatial WM involves a widespread activation of the right-lateralised dlPFC, anterior cingulate, superior temporal gyrus, interoccipital sulcus, basal ganglia and the bilateral anterior insula [128]. To investigate potential differences between verbal and visuospatial WM processes on a neural level in AN, the n-back task would be highly suitable, as it can be modified to an auditory or a visual version, and thus allow differentiation between WM components within patients [29]. Lycke et al. [129] coupled this approach with functional magnetic resonance imaging (fMRI) in a healthy sample. Interestingly, the phonological n-back elicited a left-lateralised activation, while also recruiting the frontal cortex to a greater extent than the visuospatial n-back [129]. As AN patients have been suggested to have a reduced resting-state functional connectivity of the inferior frontal gyrus, the location of Broca’s area, they may be less able to employ this region for phonological WM tasks [130].

Moreover, neural activity patterns underlying inner speech in WM have been explored by instructing participants to silently rehearse alphabetical letters [131]. In contrast to the storage condition, the manipulation of information using inner speech increased neural activity in the inferior frontal cortex and the cerebellum [131]. What further adds to the complexity of internalised vocalisation, is that monologic and dialogic inner speech may elicit different activity patterns, as seen in participants asked to generate internal self-talk in response to scenarios during fMRI [132]. A difference between monologic and dialogic inner speech may be of importance in AN, as the anorexic voice could be experienced as second- or third-person commentary [23]. Similar research in AN patients is lacking, but prospective studies could offer neuroanatomical insights into the anorexic voice and potential overlaps with WM processes.

### Future directions and implications

The difficulties in neuropsychological functioning that may arise in AN are becoming increasingly characterised (e.g. [133]). Inevitably, this has prompted re-evaluation of treatments, such as family therapy for adolescents and cognitive behavioural therapy for adults, which are cognitively demanding [134]. To bridge the gap between readiness-for-treatment and treatment itself, Davies and Tchanturia [134] adapted cognitive strategies used to alter thinking processes in psychiatric disorders to create an AN-focused cognitive remediation therapy (CRT). The overall goal of CRT is to enhance awareness of rigid cognitions, independently of ED-related cognitions, which in turn benefit the participation in primary therapies [136]. Although CRT for AN targets set shifting and central coherence deficits, there is support for the targeting of WM as well within such therapies [73, 137, 138], particularly according to this review, within the phonological (verbal/auditory) domain. Likewise, WM may be targeted in attention bias modification training aiming to re-evaluate AN-salient stimuli such as food [138]. Changing how attention is allocated in AN, could prevent the updating of pathological ruminations held in WM and allow new associations, e.g., viewing food without fear, to be rehearsed [138].

The findings of the current review invite experimental approaches to test whether phonological WM is indeed impaired in AN. For example, dual tasking has been successful in reducing the emotionality of auditory hallucinations [140]. By manipulating the active conditions, e.g., auditory taxation (counting aloud) or visual taxation (eye movements) while performing a task, the methodology could help with pinpointing any modality-specific deficits in AN cohorts. If an anorexic voice acts as hijacker of the phonological loop, future intervention strategies could involve firstly acknowledging the repetitive critical commentary and then work to restructure the maladaptive tone of the voice [22, 141]. However, the inherent subvocal nature of this proposed phenomena makes it difficult to systematically capture [86]. An initial step in resolving this could be through the recently developed Experience of an Anorexic VoicE Questionnaire (EAVE-Q) [142]. Although the early validity and internal consistency measures of the EAVE-Q have been promising, further re-tests will be necessary to fully determine its reliability. Moreover, it would be intriguing to examine the relationship between verbal/auditory WM performance and EAVE-Q scores before and after a period of exercises aimed to enhance the phonological loop and treat disorder-related inner speech.

### Strength and limits

This systematic review emphasises that there are still elements of WM capacity that need further consideration in the context the development and maintenance of AN. Importantly, we stress that modality-specific approaches when measuring WM in AN are needed, rather than combining phonological and visuospatial results. We also considered the separate age groups of patients in the reviewed papers, which revealed that deficits in WM may go undetected when adolescents and adults with AN are grouped together. The number of verbal/auditory WM tasks was underpowered in the reviewed publications, which limits the conclusions that can be drawn and justifies further experimental attention on the nature of WM in AN. Interestingly, the auditory n-back was not used in any of the reviewed studies despite being a standard WM measure [143, 144]. We must also consider that neuropsychological tests often assess several domains of executive functioning, which is why we must be cautious about the specificity of the detected impairments [145]. The review did not include intelligent quotient (IQ) as an outcome measure, as nine of the studies included in the main analysis did not detect a statistical significant difference between the AN and HC groups [40, 55, 56, 59, 60, 62, 72, 75, 76] (Online Resource Table S5). Six of the studies did not use an IQ measure [42, 43, 57, 71, 73, 74], three studies IQ-matched HC and AN patients [39, 58, 61], while only one found differences in verbal and non-verbal IQ in AN patients [18]. However, IQ has been suggested to be above average in individuals with AN and is closely related to WM, which makes it an outcome of interest for this research area [146, 147]. In addition, incorporating fMRI techniques during WM task performance (e.g. [129, 131]) would provide further insights into the active brain regions and underlying neural mechanisms than underpin EDs. Another concern is that some of the reviewed studies did not report or control for medication use and psychiatric comorbidities. As 70% of individuals with an ED are also burdened by comorbidities, the confounding effects of other disorders and pharmacological treatments against these, e.g., antidepressants and antipsychotics, need to be examined as well [148]. Furthermore, the majority of the included studies had all-female participant groups, which limits the generalizability of our findings to the male population.

## Conclusion

This study is, to our knowledge, the first to review verbal/auditory and visuospatial WM as separate domains in individuals with AN. When WM is examined globally in mixed ages of AN patients, only a minority of studies exhibit deficits. However, when adolescents and adults are tested separately in each of the two WM subsystems, it appears that the phonological domain is selectively impaired. This could be the consequence of an anorexic voice disturbing rehearsal processes essential for a healthier, non-ED lifestyle. Thus, the novel findings of this systematic review emphasise that WM as a cognitive marker for AN should not be overlooked. Instead, future research would benefit from distinguishing the WM subsystems and addressing the complex interplay between AN subtypes, duration of illness and accompanying comorbidities.

**What is already known on this subject?**

Research is divided regarding if working memory is influenced in anorexia. Cognitive deficits are becoming increasingly characterised, but the high frequency of comorbidities makes this challenging.

**What does this study add?**

The results indicated that analysing working memory subsystems and age groups separately, should be considered prerequisites for disentangling anorexia-related alterations in working memory.

## Supplementary Information

Below is the link to the electronic supplementary material.

Supplementary file1 (DOCX 20 KB)

Supplementary file2 (DOCX 24 KB)

Supplementary file3 (DOCX 20 KB)

Supplementary file4 (DOCX 19 KB)

Supplementary file5 (DOCX 19 KB)
