# Supplementary material for: Phonological working memory is adversely affected in adults with anorexia nervosa: a systematic literature review
Source: Eat Weight Disord. 2022 Feb 8;27(6):1931–52. doi: 10.1007/s40519-022-01370-1 (PMC9287223; doi:10.1007/s40519-022-01370-1)
Supplement: Supplementary file 2 — Supplementary file2 (DOCX 24 KB) [file 40519_2022_1370_MOESM2_ESM.docx]

| **Supplementary Table S2:** Risk of bias assessment (AXIS tool) | | | | | | | | | | | | | | | | | | | | | | | | | |  |
| --- | --- | --- | --- | --- | --- | --- | --- | --- | --- | --- | --- | --- | --- | --- | --- | --- | --- | --- | --- | --- | --- | --- | --- | --- | --- | --- |
|  | Tenconi et al. (2021) | Konstantakopoulos et al. (2020a) | Konstantakopoulos et al. (2020b) | Terhoeven et al. (2021) | Seidel et al. (2021) | Ogata et al. (2021) | Terhoeven et al. (2017) | Malagoli et al. (2020) | Foerde et al. (2017) | Vicario et al. (2018) | Kjærsdam et al. (2016) | van Noort et al. (2016) | Zegarra-Valdivia et al. (2018) | Kucharska et al. (2019) | Tseng et al. (2017) | Cavalera et al. (2018) | Harper et al. (2017) | Gagnon et al. (2018) | Brockmeyer et al. (2018) | Rylander et al. (2020) | Øverås et al. (2017) | Cipolletta et al. (2017) | Natalia et al. (2017) | Tamiya et al. (2018) | Cholet et al. (2021) | |
| **Introduction** | | | | | | | | | | | | | | | | | | | | | | | | | |  |
| 1. Were the aims/objectives of the study clear? | Yes | Yes | Yes | Yes | Yes | Yes | Yes | Yes | Yes | Yes | Yes | Yes | Yes | Yes | Yes | Yes | Yes | Yes | Yes | Yes | Yes | Yes | Yes | Yes | Yes | |
| **Methods** | | | | | | | | | | | | | | | | | | | | | | | | | |  |
| 2. Was the study design appropriate for the stated aim(s)? | Yes | Yes | Yes | Yes | Yes | Yes | Yes | Yes | Yes | Yes | Yes | Yes | Yes | Yes | Yes | Yes | Yes | Yes | Yes | Yes | Yes | Yes | Yes | Yes | Yes | |
| 3. Was the sample size justified? | Yes | No | Yes | No | No | Yes | No | No | Yes | Yes | Yes | No | No | Yes | No | No | Yes | No | Yes | Yes | Yes | No | No | No | No | |
| 4. Was the target/reference population clearly defined? (Is it clear who the research was about?) | Yes | Yes | Yes | Yes | Yes | Yes | Yes | Yes | Yes | Yes | Yes | Yes | Yes | Yes | Yes | Yes | Yes | Yes | Yes | Yes | Yes | Yes | Yes | Yes | Yes | |
| 5. Was the sample frame taken from an appropriate  population base so that it closely represented the  target/reference population under investigation? | Yes | Yes | Yes | Yes | Yes | Yes | Yes | Yes | Yes | Yes | Yes | Yes | ND | Yes | Yes | Yes | Yes | Yes | Yes | Yes | Yes | Yes | Yes | Yes | Yes | |
| 6. Was the selection process likely to select subjects/participants that were representative of the target/reference population under investigation? | Yes | Yes | Yes | Yes | Yes | Yes | Yes | Yes | Yes | Yes | Yes | Yes | ND | Yes | Yes | Yes | Yes | Yes | Yes | Yes | Yes | Yes | Yes | Yes | Yes | |
| 7. Were measures undertaken to address and categorize non-responders? | ND | ND | Yes | ND | ND | ND | ND | ND | Yes | ND | Yes | ND | ND | ND | ND | ND | Yes | ND | ND | ND | ND | ND | ND | ND | ND | |
| 8. Were the risk factor and outcome variables measured appropriate to the aims of the study? | Yes | Yes | Yes | Yes | Yes | Yes | Yes | Yes | Yes | Yes | Yes | Yes | Yes | Yes | Yes | Yes | Yes | Yes | Yes | Yes | Yes | Yes | Yes | Yes | Yes | |
| 9. Were the risk factor and outcome variables measured correctly using instruments/ measurements that had been trialled, piloted or published previously? | Yes | Yes | Yes | Yes | Yes | Yes | Yes | Yes | Yes | Yes | Yes | Yes | Yes | Yes | Yes | Yes | Yes | Yes | Yes | Yes | Yes | Yes | Yes | Yes | Yes | |
| 10. Is it clear what was used to determined statistical significance and/or precision estimates? (e.g., p values, CIs) | Yes | Yes | Yes | Yes | Yes | Yes | Yes | Yes | Yes | Yes | Yes | Yes | Yes | Yes | Yes | Yes | Yes | Yes | No | Yes | Yes | Yes | Yes | Yes | Yes | |
| 11. Were the methods (including statistical methods) sufficiently described to enable them to be repeated? | Yes | Yes | Yes | Yes | Yes | Yes | Yes | Yes | Yes | Yes | Yes | Yes | Yes | Yes | Yes | Yes | Yes | Yes | No | Yes | Yes | Yes | Yes | Yes | Yes | |
| **Results** | | | | | | | | | | | | | | | | | | | | | | | | | |  |
| 12. Were the basic data adequately described? | Yes | Yes | Yes | Yes | Yes | Yes | Yes | No | Yes | Yes | No | Yes | No | Yes | Yes | No | Yes | Yes | Yes | Yes | Yes | Yes | Yes | Yes | Yes | |
| 13. Does the response rate raise concerns about nonresponse bias? | ND | ND | No | ND | ND | ND | ND | ND | No | ND | ND | ND | ND | ND | ND | ND | No | ND | ND | ND | ND | ND | ND | ND | ND | |
| 14. If appropriate, was information about nonresponders described? | No | No | No | No | No | No | No | No | Yes | No | No | No | No | No | No | No | Yes | No | No | No | No | No | No | No | No | |
| 15. Were the results internally consistent? | Yes | Yes | Yes | Yes | Yes | Yes | Yes | Yes | Yes | Yes | Yes | Yes | Yes | Yes | Yes | Yes | Yes | Yes | Yes | Yes | Yes | Yes | Yes | Yes | Yes | |
| 16. Were the results for the analyses described in the methods, presented? | Yes | Yes | Yes | Yes | Yes | Yes | Yes | Yes | Yes | Yes | Yes | Yes | Yes | Yes | Yes | Yes | Yes | Yes | Yes | Yes | Yes | Yes | Yes | Yes | Yes | |
| **Discussion** | | | | | | | | | | | | | | | | | | | | | | | | | |  |
| 17. Were the authors’ discussions and conclusions justified by the results? | Yes | Yes | Yes | Yes | Yes | Yes | Yes | Yes | Yes | Yes | Yes | Yes | Yes | Yes | Yes | Yes | Yes | Yes | Yes | Yes | Yes | Yes | Yes | Yes | Yes | |
| 18. Were the limitations of the study discussed? | No | Yes | Yes | Yes | Yes | Yes | Yes | Yes | Yes | Yes | Yes | Yes | Yes | Yes | Yes | Yes | Yes | Yes | Yes | Yes | Yes | Yes | Yes | Yes | Yes | |
| **Others** | | | | | | | | | | | | | | | | | | | | | | | | | |  |
| 19. Were there any funding sources or conflicts of  interest that may affect the authors’ interpretation of  the results? | No | No | No | No | Yes | No | No | No | No | No | ND | No | No | No | No | ND | ND | No | No | No | ND | No | ND | No | No | |
| 20. Was ethical approval or consent of participants attained? | Yes | Yes | Yes | Yes | Yes | Yes | Yes | Yes | Yes | Yes | Yes | Yes | Yes | Yes | Yes | Yes | Yes | Yes | Yes | Yes | Yes | Yes | Yes | Yes | Yes | |
| Not described (ND)  Downes, M.J.; Brennan, M.L.; Williams, H.C.; Dean, R.S. Development of a critical appraisal tool to assess the quality of cross-sectional studies (AXIS). BMJ Open 2016, 6,  e011458, doi:10.1136/bmjopen-2016-011458. | | | | | | | | | | | | | | | | | | | | | | | | | |  |

Title: Phonological working memory is adversely affected in adults with anorexia nervosa: a systematic literature review

Journal: *Eating and Weight Disorders - Studies on Anorexia, Bulimia and Obesity*

Authors: Amelia D. Dahlén^*a^, Santino Gaudio, Helgi B. Schiöth and Samantha J. Brooks*^a,b,c^

*Corresponding authors: dahlenamelia@gmail.com, S.J.Brooks@ljmu.ac.uk

^a^Section of Functional Pharmacology, Department of Neuroscience, Uppsala University, 75124 Uppsala, Sweden

^b^School of Psychology, Faculty of Health, Liverpool John Moores University, Liverpool, United Kingdom

^c^Neuroscience Research Laboratory (NeuRL), Department of Psychology, School of Human and Community Development, University of the Witwatersrand, Johannesburg, South Africa
