# Supplementary material for: Phonological working memory is adversely affected in adults with anorexia nervosa: a systematic literature review
Source: Eat Weight Disord. 2022 Feb 8;27(6):1931–52. doi: 10.1007/s40519-022-01370-1 (PMC9287223; doi:10.1007/s40519-022-01370-1)
Supplement: Supplementary file 3 — Supplementary file3 (DOCX 20 KB) [file 40519_2022_1370_MOESM3_ESM.docx]

| **Table S3.** Verbal/Auditory working memory performance in participants with anorexia nervosa (AN) in comparison to healthy controls (HC) | | | | | | | | |
| --- | --- | --- | --- | --- | --- | --- | --- | --- |
| **Arithmetic** | **Digit Span** | **LNS** | **RAVLT/ VLMT** | **RSPAN** | **Story recall** | **WM Index** | **Numeric WM** | **Immediate word recall** |
| Tseng et al. (2017) *ED + Bipolar < ED + MDD  **DEFICIT** ADULTS | Zegarra-Valdivia et al. (2018)  **DEFICIT**  ADOLESCENTS | Tamiya et al. (2018)  **NO DIFFERENCE** MIXED AGES | Terhoeven et al. (2017)  **DEFICIT**  ADULTS | Malagoli et al. (2020) *medicated ED group  **DEFICIT** MIXED AGES | Terhoeven et al. (2017)  **DEFICIT**  ADULTS | Ogata et al. (2021) (AN-R vs. HC)  **NO DIFFERENCE**  ADULTS | Seidel et al. (2021)  **NO DIFFERENCE**  ADULTS | Seidel et al. (2021)  **NO DIFFERENCE** ADULTS |
|  |  |  |  |  |  |  |  |  |
|  | Konstantakopoulos et al. (2020a)  **DEFICIT** | Foerde & Steinglass (2017)  **NO DIFFERENCE** | Natalia et al. (2017)  **DEFICIT** |  |  |  |  |  |
|  | ADULTS | MIXED AGES | MIXED AGES |  |  |  |  |  |
|  |  |  |  |  |  |  |  |  |
|  | Konstantakopoulos et al. (2020b) |  |  |  |  |  |  |  |
|  | **DEFICIT** |  |  |  |  |  |  |  |
|  | ADULTS |  |  |  |  |  |  |  |
|  |  |  |  |  |  |  |  |  |
|  | Tseng et al. (2017) *ED + Bipolar < ED + MDD |  |  |  |  |  |  |  |
|  | **DEFICIT** |  |  |  |  |  |  |  |
|  | ADULTS |  |  |  |  |  |  |  |
|  |  |  |  |  |  |  |  |  |
|  | Brockmeyer et al. (2018) |  |  |  |  |  |  |  |
|  | **NO DIFFERENCE** |  |  |  |  |  |  |  |
|  | ADULTS |  |  |  |  |  |  |  |
|  |  |  |  |  |  |  |  |  |
|  | Cholet et al. (2020)  (AN-R vs. HC) |  |  |  |  |  |  |  |
|  | **NO DIFFERENCE** |  |  |  |  |  |  |  |
|  | ADULTS |  |  |  |  |  |  |  |
|  |  |  |  |  |  |  |  |  |
|  | Gagnon et al. (2018) |  |  |  |  |  |  |  |
|  | **NO DIFFERENCE** |  |  |  |  |  |  |  |
|  | ADULTS |  |  |  |  |  |  |  |
|  |  |  |  |  |  |  |  |  |
|  | Terhoeven et al. (2021) |  |  |  |  |  |  |  |
|  | **NO DIFFERENCE**  ADULTS |  |  |  |  |  |  |  |
|  |  |  |  |  |  |  |  |  |
|  | Natalia et al. (2017)  **NO DIFFERENCE** |  |  |  |  |  |  |  |
|  | MIXED AGES |  |  |  |  |  |  |  |

**Abbreviations:** anorexia nervosa (AN), anorexia nervosa restricting subtype (AN-R), eating disorders (ED), healthy controls (HC), Letter Number Sequencing (LNS), major depressive disorder (MDD), Reading Span task (RSPAN), Rey Auditory Verbal Learning Test (RAVLT), Verbal Learning and Memory Test (VLMT), Working memory (WM).

Title: Phonological working memory is adversely affected in adults with anorexia nervosa: a systematic literature review

Journal: *Eating and Weight Disorders - Studies on Anorexia, Bulimia and Obesity*

Authors: Amelia D. Dahlén^*a^, Santino Gaudio, Helgi B. Schiöth and Samantha J. Brooks*^a,b,c^

*Corresponding authors: dahlenamelia@gmail.com, S.J.Brooks@ljmu.ac.uk

^a^Section of Functional Pharmacology, Department of Neuroscience, Uppsala University, 75124 Uppsala, Sweden

^b^School of Psychology, Faculty of Health, Liverpool John Moores University, Liverpool, United Kingdom

^c^Neuroscience Research Laboratory (NeuRL), Department of Psychology, School of Human and Community Development, University of the Witwatersrand, Johannesburg, South Africa
