# Supplementary material for: Phonological working memory is adversely affected in adults with anorexia nervosa: a systematic literature review
Source: Eat Weight Disord. 2022 Feb 8;27(6):1931–52. doi: 10.1007/s40519-022-01370-1 (PMC9287223; doi:10.1007/s40519-022-01370-1)
Supplement: Supplementary file 4 — Supplementary file4 (DOCX 19 KB) [file 40519_2022_1370_MOESM4_ESM.docx]

| **Table S4.** Visuospatial working memory performance in participants with anorexia nervosa (AN) in comparison to healthy controls (HC) | | | | | | | |
| --- | --- | --- | --- | --- | --- | --- | --- |
| **Corsi/Spatial Span** | **Digit Span** | **N-back** | **RCFT** | **SDMT** | **SymmSpan** | **Spatial WM** | **TMT-B/condition 4** |
| Natalia et al. (2017) (backward)  **DEFICIT**  MIXED AGES | Vicario & Felmingham (2018)  **NO DIFFERENCE** ADOLESCENTS | Natalia et al. (2017) (AN-R<HC)  **DEFICIT**  MIXED AGES | Cholet et al. (2020) (AN-R > HC)  **BETTER**  ADULTS | Natalia et al. (2017) (AN-R< HC)  **DEFICIT**  MIXED AGES | Malagoli et al. (2020) *medicated ED  **DEFICIT** | Seidel et al. (2021)  **NO DIFFERENCE**  ADULTS | Kucharska et al. (2019)  **DEFICIT**  ADOLESCENTS |
|  |  |  |  |  | MIXED AGES |  | Natalia et al. (2017) |
| Cipolletta et al. (2017) |  | Natalia et al. (2017) **NO DIFFERENCE** | Øverås et al. (2017)  **NO DIFFERENCE** | Natalia et al. (2017)  **NO DIFFERENCE** |  |  | **DEFICIT**  MIXED AGES |
| **NO DIFFERENCE** |  | MIXED AGES | ADULTS | MIXED AGES |  |  |  |
| MIXED AGES |  |  |  |  |  |  | Terhoeven et al. (2021)  **DEFICIT** |
| Natalia et al. (2017) (forward) |  |  | Natalia et al. (2017)  **NO DIFFERENCE** |  |  |  | ADULTS |
| **NO DIFFERENCE** MIXED AGES |  |  | MIXED AGES |  |  |  | Foerde & Steinglass (2017) |
| Tamiya et al. (2018) |  |  | van Noort et al. (2016) |  |  |  | **NO DIFFERENCE** MIXED AGES |
| **NO DIFFERENCE** |  |  | **NO DIFFERENCE** |  |  |  |  |
| MIXED AGES |  |  | ADOLESCENTS |  |  |  | van Noort et al. (2016) |
|  |  |  |  |  |  |  | **NO DIFFERENCE** |
| Gagnon et al. (2018) **NO DIFFERENCE** |  |  | Kjærsdam Telléus et al. (2016) |  |  |  | ADOLESCENTS |
| ADULTS |  |  | **NO DIFFERENCE** ADOLESCENTS |  |  |  | Vicario & Felmingham (2018) |
|  |  |  |  |  |  |  | **NO DIFFERENCE** |
|  |  |  | Tanconi et al. (2021) |  |  |  | ADOLESCENTS |
|  |  |  | **DEFICIT**  MIXED AGES |  |  |  | Kjærsdam Telléus et al. (2016) |
|  |  |  |  |  |  |  | **NO DIFFERENCE** |
|  |  |  |  |  |  |  | ADOLESCENTS |
|  |  |  |  |  |  |  |  |
|  |  |  |  |  |  |  | Gagnon et al. (2018)  **NO DIFFERENCE** |
|  |  |  |  |  |  |  | ADULTS |
|  |  |  |  |  |  |  |  |
|  |  |  |  |  |  |  | Terhoeven et al. (2017)  **NO DIFFERENCE** |
|  |  |  |  |  |  |  | ADULTS |
|  |  |  |  |  |  |  |  |
|  |  |  |  |  |  |  | Rylander et al. (2020)  **NO DIFFERENCE** |
|  |  |  |  |  |  |  | ADULTS |

**Abbreviations:** anorexia nervosa (AN), anorexia nervosa restricting subtype (AN-R), eating disorders (ED), healthy controls (HC), Rey Complex Figure Test (RCFT), Symbol-Digit Modalities Test (SDMT), Symmetry span task (SymmSpan), Trail Making Test (TMT).

Title: Phonological working memory is adversely affected in adults with anorexia nervosa: a systematic literature review

Journal: *Eating and Weight Disorders - Studies on Anorexia, Bulimia and Obesity*

Authors: Amelia D. Dahlén^*a^, Santino Gaudio, Helgi B. Schiöth and Samantha J. Brooks*^a,b,c^

*Corresponding authors: dahlenamelia@gmail.com, S.J.Brooks@ljmu.ac.uk

^a^Section of Functional Pharmacology, Department of Neuroscience, Uppsala University, 75124 Uppsala, Sweden

^b^School of Psychology, Faculty of Health, Liverpool John Moores University, Liverpool, United Kingdom

^c^Neuroscience Research Laboratory (NeuRL), Department of Psychology, School of Human and Community Development, University of the Witwatersrand, Johannesburg, South Africa
