# Supplementary material for: Phonological working memory is adversely affected in adults with anorexia nervosa: a systematic literature review
Source: Eat Weight Disord. 2022 Feb 8;27(6):1931–52. doi: 10.1007/s40519-022-01370-1 (PMC9287223; doi:10.1007/s40519-022-01370-1)
Supplement: Supplementary file 5 — Supplementary file5 (DOCX 19 KB) [file 40519_2022_1370_MOESM5_ESM.docx]

**a**
